# An Overview of the Medical Applications of Marine Skeletal Matrix Proteins

**DOI:** 10.3390/md14090167

**Published:** 2016-09-12

**Authors:** M. Azizur Rahman

**Affiliations:** 1 Department of Chemical & Physical Sciences, University of Toronto Mississauga, Mississauga, ON L5L 1C6, Canada; mazizur.rahman@utoronto.ca or azizurr142@gmail.com; Tel.: +1-647-892-4221; 2 HiGarden Inc., Markham, ON L3R 3W4, Canada

**Keywords:** biomineralization, coralline algae, chitin, collagen, marine calcifiers, marine skeletal proteins, proteomics

## Abstract

In recent years, the medicinal potential of marine organisms has attracted increasing attention. This is due to their immense diversity and adaptation to unique ecological niches that has led to vast physiological and biochemical diversification. Among these organisms, marine calcifiers are an abundant source of novel proteins and chemical entities that can be used for drug discovery. Studies of the skeletal organic matrix proteins of marine calcifiers have focused on biomedical applications such as the identification of growth inducing proteins that can be used for bone regeneration, for example, 2/4 bone morphogenic proteins (BMP). Although a few reports on the functions of proteins derived from marine calcifiers can be found in the literature, marine calcifiers themselves remain an untapped source of proteins for the development of innovative pharmaceuticals. Following an overview of the current knowledge of skeletal organic matrix proteins from marine calcifiers, this review will focus on various aspects of marine skeletal protein research including sources, biosynthesis, structures, and possible strategies for chemical or physical modification. Special attention will be given to potential medical applications and recent discoveries of skeletal proteins and polysaccharides with biologically appealing characteristics. In addition, I will introduce an effective protocol for sample preparation and protein purification that includes isolation technology for biopolymers (of both soluble and insoluble organic matrices) from coralline algae. These algae are a widespread but poorly studied group of shallow marine calcifiers that have great potential for marine drug discovery.

## 1. Introduction

Skeletal proteins and polysaccharides in marine organisms are present as complex mixtures within organic matrices. The organic matrices of marine calcifiers, for example, are a potentially untapped source of skeletal proteins [1,2,3,4,5,6]. Organic matrices have the advantage of being naturally produced, retaining the native, functional conformation of the original proteins. Moreover, a significant number of calcifying marine invertebrates produce polysaccharides within their extracellular matrices and connective tissues [7,8] that have molecular structures and functions similar to human versions [1,6]. Polysaccharides derived from marine invertebrate extracellular matrices encompass an enormous variety of structures and should be considered as an extraordinary source of biochemical diversity. However, they remain largely under-exploited with respect their potential in medical applications [9,10]. Macromolecules derived from marine calcifiers that hold promise for biomedical applications include a broad range of protein and sugar (carbohydrates and lectins) molecules that participate in signaling, development, regeneration, and metabolism.

It is has been hypothesized that marine skeletal proteins that function in biomineral growth, maintenance, and repair could facilitate tissue engineering. For example, some of these proteins with human physiological activity can help accelerate lab-based bone morphogenesis and increase bone volumes with efficacies equivalent to currently used recombinant proteins [1]. Proteins with potential for bone repair and drug discovery, extracted either from naturally occurring skeletal organic matrices or derived from cultivated tissues, can be identified and isolated using chromatography, cell assays and proteomic methods [1,9,11]. Proteomics is a high-throughput analytical method for rapidly identifying known or unknown proteins in complex mixtures [5]. If purification methods can be established for skeletal proteins derived from calcifying marine organisms, researchers in the emerging fields of proteomics and medicinal chemistry could utilize these methods for subsequent drug discovery and, as a more specific example, bone repair. Currently, primary sequences of different skeletal proteins from marine organisms are available in public databases, and this information can be used to infer the biological function and origin of individual proteins and provide clues related to the mechanisms of formation of any skeleton.

Pharmaceutical industries now accept the world’s oceans as a major frontier for medical research. The emergence of this relatively new area of scientific exploration has been of enormous interest to the popular and scientific press, and several review publications have appeared on the topic [1,9,11]. In the review presented here, we focus on recent progress in the discovery and production of new marine skeletal proteins and polysaccharides of pharmaceutical interest. We also introduce a new technique for purifying compounds derived from the skeletal organic matrices of coralline algae that will be useful for proteomic analysis and purifying biopolymers such as chitin and collagen. Overall, this review demonstrates the existence of unique biomineralization-related skeletal proteins in marine calcifiers that hold promise for drug development, and moreover, provides the first description of proteinaceous components in coralline red algae.

## 2. Applications and Modification Strategies of Marine Skeletal Proteins for Drug Discovery

Marine calcifiers (shallow, mid-shelf, and deep sea) are widespread in oceans globally. However, due to the lack of effective extraction/analytical methods, the applications of these potential resources for drugs are comparatively fewer than for other marine organisms. Recently, we perceived protein induced crystallization [2,7,8,12,13], which showed potential crystal design and growth that could help medicinal chemistry in drug design. Our primary chemical proteomic results from soft coral revealed a number of molecules with high concentrations [5,14]. In addition, some proteins extracted from soft corals are homologous with many human proteins, making them useful due to their similarity [15,16]. The information with respect to the close homology of soft coral and human proteins provides us functional and evolutionary clues on the structure and functions of their sequences. These homologous proteins could lead to possible drug discovery and form a potential resource for biotechnological research. It is our hope that further sequence studies of these materials will contribute to a better understanding of structural proteins in soft corals. Bioassay-directed fractionation of octocoral *Cespitularia hypotentaculata*, which has a novel endoskeleton, yielded the diterpene cespitularin A–D, the norditerpene cespitularin E and three other diterpenes, cespitularin F–H [17]. Two new dolabellane-type diterpenoids and the known diterpene clavenone [18] were isolated from a octocoral *Clavularia* species [19]. A saponin compound was isolated from the octocoral *Lobophytum* spp., which was collected from Hainan Island, China.

Among the marine calcifiers, very few scleractinian corals were investigated. In a recent review, the authors discussed the potential of scleractinian coral, which has therapeutic characteristics, including anti-inflammatory properties, anticancer properties, bone repair, and neurological benefits [6]. Research on the scleractinian coral *Montipora* spp. from the republic of Korea (South Korea) found three diacetylenes (1, 4, 6). One of these was a potent cytotoxin with respect to a range of tumor cell lines [20]. The authors tested the extracted compounds against a panel of human cancer cell lines and the structures have been interpreted on the basis of spectroscopic evidence. These three compounds showed a structural activity profile to similar to those previously reported [21]. The results showed that the compound 6 with b-hydroxy ketone functionality has strong cytotoxic properties and Methyl montiporate C (1) was active only against a skin cancer cell line, while compound 4 was moderately active. Extracts from the calcifying octocorals *Pseudopterogorgia elizabethae* (which contains pseudopterosins) and *Eunicea fusca* (which contains fucoside-A) can be used in the cosmetic industry [22]. Similarly, coral (endoskeletons and exoskeletons) and coralline algal skeletons could be used for cosmetics as both contain a high concentration of organic matrix components [7,13,23].

In recent years, numerous applications have been proposed for chitosan-based delivery devices [24,25,26], however, most of these were unrelated to marine calcifiers. Chitosan is a copolymer of β-(1-4)-linked 2-acetamido-2-deoxy-d-glucopyranose and 2-amino-2-deoxy-d-glucopyranose, obtained by deacethylation of the naturally occurring chitin. Chitin was firstly extracted from the exoskeleton of marine organisms, mainly crabs and shrimps, as described by Burrows [27]. This polymer has also recently been extracted from coralline algae [7], which opened the doors for possible applications of these biomaterials using a group of marine calcifers which are found in shallow water and are easy-to-collect, abundant and widespread. The major applications of chitosan are for biomaterials, pharmaceuticals, foodstuff treatment (e.g., flocculation, clarification, etc., due to its efficient interaction with other polyelectrolytes), cosmetics, metal ion sequestration, and agriculture [28,29,30,31]. Development of chitosan chemistry has relevant biomedical applications, particularly in the field of drug delivery [32]. While chitin is insoluble in most common solvents, chitosan can be readily turned into fibers as well as films, or triggered in a variety of micromorphologies from its acidic aqueous solutions. Protein-polysaccharides play an important role in biomedical and pharmaceutical applications. However, at times the properties of such biomaterials do not meet the needs for exact applications. As a result, approaches that chemically or physically modify their structure and, thus, physical-chemical properties are increasingly gaining interest [33,34]. With respect to the polysaccharides’ chitin and chitosan, it is possible to target the reaction using sulfur trioxide-pyridine at two sites or at only one specific site, following different pathways of synthesis [35]. Great efforts have thus focused on the progress of efficient modification reactions in well-controlled conditions under tolerable temperatures [35]. For example, modification reactions of water-soluble chitin can be conducted in aqueous solutions or in organic solvents in an engorged state under mild conditions, and selective *N*-acetylation [35]. Some significant chemical reactions of acylation, alkylation, Schiff base formation and reductive *N*-alkylation, carboxyalkylation, *N*-phthaloylation are well described [35].

## 3. A Promising Future for Marine Calcifiers in Drug Discovery

Marine resources such as coral, mollusk and coralline algae could be a major source of medicines over the next decades. It is estimated that marine ecosystems, such as those found in coral reefs or at a deep sea level have greater biological diversity than those of tropical rain forests. However, as with tropical rain forests, coral reefs represent considerable untouched potential in the science of medicine.

At present, marine calcifier collection and drug appraisal occurs successfully. However, there is no question that these resources are inadequate and it is possible that collectable marine organisms will be almost completely explored within the next 20 years. There is still a doubt as to where scientists will turn in order to ensure a continuing flow of new medicines. The solution is difficult, however drugs can now be developed using many methods such as computer-aided design, combinatorial synthesis and proteomics. The chemical multiplicity of marine ecosystems, from simple to complex peptide and protein extraction, draws us in the direction of the discovery of new marine natural products in various therapeutic areas such as cancer, inflammation, microbial infections, and various other deadly diseases [36]. Cancer is the biggest challenge of the current century, and marine calcifying organisms show new promise in fighting against this and other dangerous diseases.

## 4. A Novel Approach to Isolation, Purification and Characterization of Marine Skeletal Proteins

**Figure 1:**
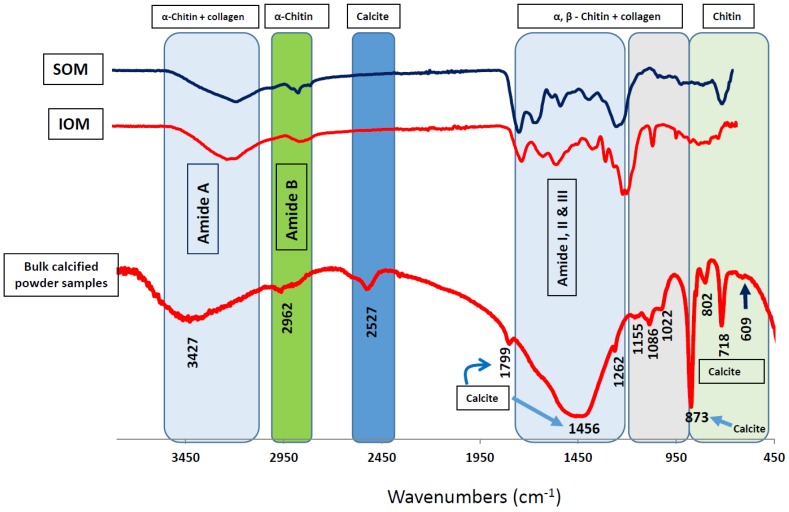
Identification of chitin and collagen in algal skeletal protein-polysaccharides complexes. Structural comparison of FTIR spectra between organic matrix fractions (soluble organic matrix (SOM) and insoluble organic matrix (IOM)) and bulk skeletal powder. Graphs for SOM, IOM fractions and bulk skeletal powder are indicated. Different colored boxes in the spectra indicate involvement of molecules in SOM and IOM fractions in forming skeletal structure in coralline algal calcification system. (Reproduced from Scientific Reports, Rahman and Halfar 2014 [7]).

Isolation and purification of skeletal proteins from marine calcifiers are complex because of the potential for contamination of the soft tissues and the high sensitivity of organic matrices to handling. However, successfully purified skeletal proteins from several groups of marine calcifiers have recently emerged [4,5,14,22,23,37]. The overview concerning marine skeletal proteins presented above allows us to understand some newly developed techniques [5,12,14,15,16,23,38,39,40] as well as useful methods for isolating and purifying skeletal proteins and proteomic analysis. Among marine calcifiers, we recently investigated coralline red algae, which has specific biological characteristics [7] and contains high concentrations of soluble organic matrix (SOM) and insoluble organic matrix (IOM) fractions. High concentrations of both chitin and collagen biopolymers are present in SOM and IOM (Figure 1). Coralline algal concentrations of SOM (0.9%) and IOM (4.5%) are significantly higher than those of other skeletal marine calcifiers such as octocorals, with SOM and IOM concentrations of 0.03% and 0.05%, respectively [5,13,15]. The highly concentrated biopolymers present in skeletal organic matrices open up the possibility for future drug development, because these two polymers are frequently utilized in drug design [24,29,30,31,41,42,43,44,45,46].

**Figure 2:**
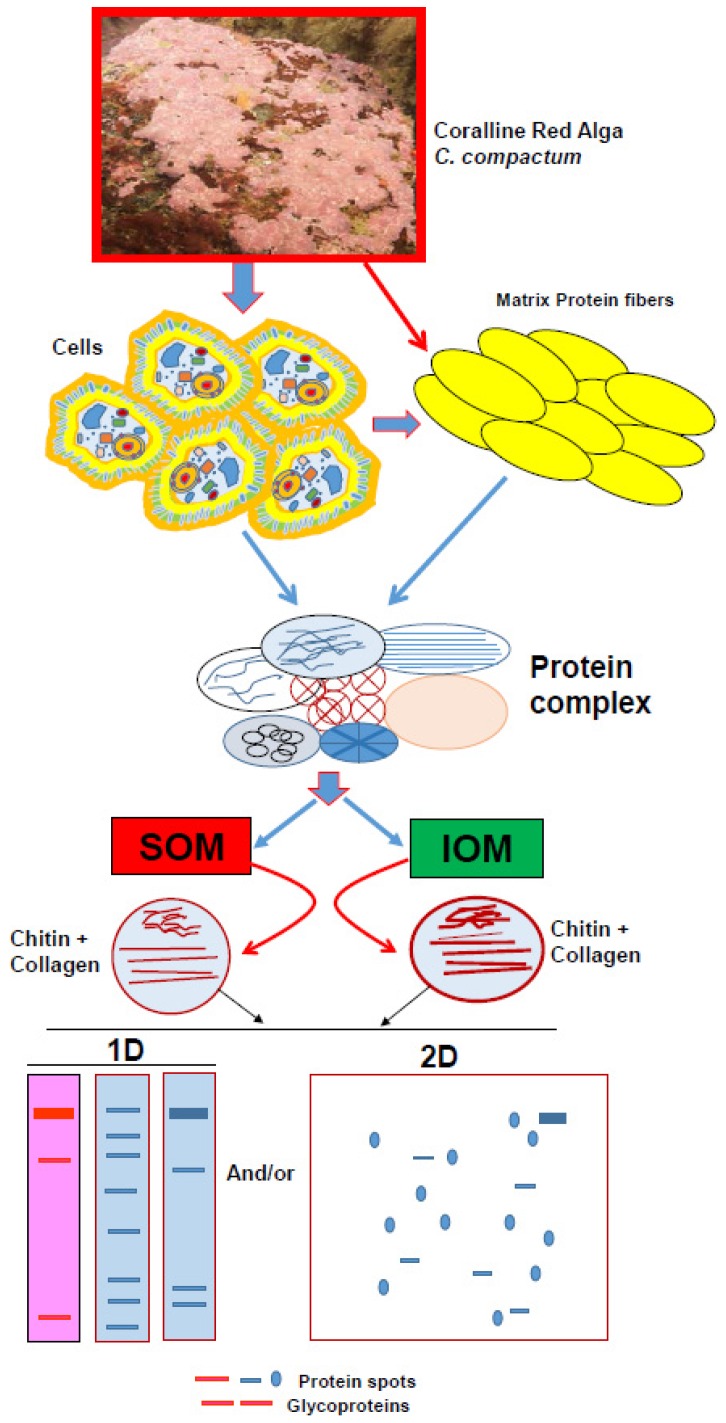
Model of general strategy for analyzing protein-polysaccharides complex from skeletal organic matrix of marine calcifiers.

**Figure 3:**
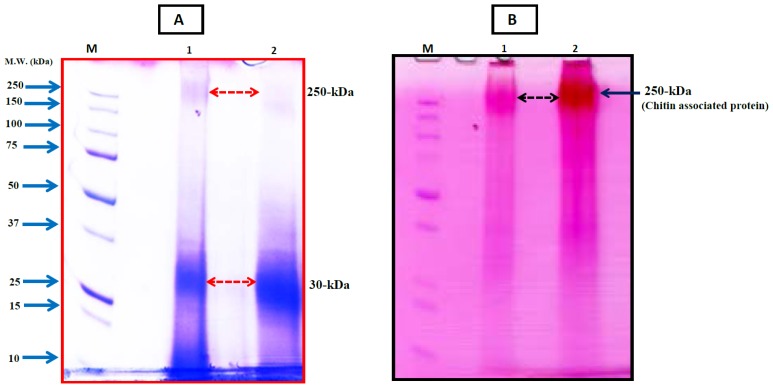
Electrophoretic analysis of skeletal matrix proteins extracted from the coralline red alga *C. compactum*. (**A**) SDS-PAGE fractionation with Coomassie Brilliant Blue (CBB) staining after purification of the skeletal proteins. Lane 1 and 2 indicate purified skeletal proteins. Arrows indicate protein bands; (**B**) SDS-PAGE gel with Periodic Acid-Schiff (PAS) staining to identify glycoprotein in skeletal matrix proteins of *C. compactum*. Lane 1 and 2, a strong abundant chitin associated glycoprotein was identified (indicated by arrow) by periodic Acid-Schiff staining. An eluate (derived from 5 g of algal skeleton) was run on 12% polyacrylamide gel M, protein marker. The Precision Plus SDS-PAGE standard (Bio-Rad) was used as protein marker for electrophoresis.

Detailed geochemical studies of coralline algae [47,48,49,50,51] provide a broad spectrum of environmental and structural background information. However, there is a lack of information on the protein-polysaccharide complex in the coralline algal skeleton, which plays a key role in the regulation of biocalcification [7] and may contain prospective biomaterials for drug development. Hence, we have developed a useful technique from sample preparation to protein isolation for the Sub-Arctic coralline alga *Clathromorphum compactum* (Figure 2, see Ref. [7] for details) using recently developed analytical approaches for other marine calcifiers (Figure 2, References [5,12,23]). We characterized the SOM-polysaccharide complex from its CaCO_3_ skeleton, which is involved in the biocalcification process. Sodium dodecyl sulfate-polyacrylamide gel electrophoresis (SDS-PAGE) analysis [52] of the preparations [5,14,23] showed two bands of proteins with molecular masses of 250-kDa and 30-kDa (Figure 3A, lane 1 and 2). The protein with molecular masses of 30-kDa was by far the most abundant protein, whereas the 250 kDa protein band was weak and somewhat faint (Figure 3A, lane 2). Periodic acid-Schiff (PAS) staining was used to identify chitin associated glycoproteins. Interestingly, the 250-kDa protein was identified with high abundance as the only glycoprotein contained in the skeleton (Figure 3B, lane 1 and 2), even though it only appeared as a weak band in Coomassie Brilliant Blue (CBB) staining solution (Figure 3A). Chitin is the main component of the protein-polysaccharide complex of cell walls [7,53,54]), which is also composed of glycoprotein [55]. Protein-polysaccharide complexes are also present in coralline algal cell structures [7]. Therefore, detection of a strong glycosylation protein in coralline algal skeletons reveals the presence of highly abundant chitin. The chitin found in coralline alga has been recognized to be involved in the calcification process [7] and this polymer is considered highly useful for drug design [24,29,30,31,41,42,43,44,45,46]. Our observations therefore strongly suggest that the skeletal matrix proteins in coralline alga are not only a structural protein but also have potential for drug development.

## 5. Conclusions

In this brief review, recent advances in applications of protein-polysaccharides of marine calcifiers in the medical and pharmaceutical fields have been discussed. The results demonstrate the potential for marine calcifiers to generate new drugs. Understanding the proteinaceous components of marine calcifiers is an important step toward advancing the science of marine medicinal chemistry. Among the different sources of polysaccharides, algal polysaccharides such as chitin and collagen could play an important role in future development of tissue engineering, bone regeneration, and much more. In light of these emerging findings, in the near future established techniques may also be potentially useful for isolating skeletal proteins from similar marine calcifiers for drug discovery. As a discovery-driven science, the techniques discussed here allow researcher to identify candidate proteins for drug discovery and identify unknowns without missing unanticipated interactions. These techniques can be employed to dramatically improve the range of applications within the field of marine drug discovery. Since the marine realm consists of diverse ecosystems and matrices in which these proteins reside, the development of effective methods for accessing proteins will be a continuing challenge in future years.
